# Lipid Corona Formation on Micro- and Nanoplastic Particles Modulates Uptake and Toxicity in A549 Cells

**DOI:** 10.3390/ma16145082

**Published:** 2023-07-19

**Authors:** Anna Daniela Dorsch, Walison Augusto da Silva Brito, Mihaela Delcea, Kristian Wende, Sander Bekeschus

**Affiliations:** 1 ZIK *plasmatis*, Leibniz Institute for Plasma Science and Technology (INP), Felix-Hausdorff-Str. 2, 17489 Greifswald, Germany; 2 Department of General Pathology, State University of Londrina, Rodovia Celso Garcia Cid, Londrina 86057-970, Brazil; 3 Institute of Biochemistry, University of Greifswald, Felix-Hausdorff-Str. 4, 17487 Greifswald, Germany; 4 Clinic and Policlinic for Dermatology and Venerology, Rostock University Medical Center, Strempelstr. 13, 18057 Rostock, Germany

**Keywords:** A549 cells, dynamic light scattering, microplastic, surface plasmon resonance, unilamellar vesicles

## Abstract

Plastic waste is a global issue leaving no continents unaffected. In the environment, ultraviolet radiation and shear forces in water and land contribute to generating micro- and nanoplastic particles (MNPP), which organisms can easily take up. Plastic particles enter the human food chain, and the accumulation of particles within the human body is expected. Crossing epithelial barriers and cellular uptake of MNPP involves the interaction of plastic particles with lipids. To this end, we generated unilamellar vesicles from POPC (1-palmitoyl-2-oleoyl-glycero-3-phosphocholine) and POPS (1-palmitoyl-2-oleoyl-sn-glycero-3-phospho-L-serine) and incubated them with pristine, carboxylated, or aminated polystyrene spheres (about 1 µm in diameter) to generate lipid coronas around the particles. Lipid coronas enhanced the average particle sizes and partially changed the MNPP zeta potential and polydispersity. In addition, lipid coronas led to significantly enhanced uptake of MNPP particles but not their cytotoxicity, as determined by flow cytometry. Finally, adding proteins to lipid corona nanoparticles further modified MNPP uptake by reducing the uptake kinetics, especially in pristine and carboxylated plastic samples. In conclusion, our study demonstrates for the first time the impact of different types of lipids on differently charged MNPP particles and the biological consequences of such modifications to better understand the potential hazards of plastic exposure.

## 1. Introduction

Plastic waste pollution in the environment has increased drastically in the past decades [1,2]. A plentitude of plastic types can be found in soils or water bodies [3,4]. Due to environmental impacts, such as ultraviolet (UV) radiation and shearing forces, the waste breaks down into micro- and nanoplastic particles (MNPP) [5]. As their numbers increase, a plentitude of particles is found in different areas of the world, even in isolated mountain regions and deep-sea sediments [6,7]. These increasing numbers of MNPP, especially in water and air, threaten (aquatic) ecosystems and even the human food chain [8,9]. There are two prevailing perspectives regarding the categorization of the term nanoplastic. Some favor the classification of particles smaller than 1000 nm, while others suggest the use of this term only for particles smaller than 100 nm [10,11,12]. An upper limit of 100 nm is commonly used by general consensus [13]. As in the environment, the size of plastic particles is likely a continuum. Therefore, it seems feasible to include both terms (microplastic and nanoplastic) in a single acronym (MNPP). The plastic particles used in this study were mostly about 1 µm.

In addition to the known formation of protein coronas around ingested nanoparticles [14,15], interactions of environmentally relevant nanoparticles with lipids [16,17,18] and modulation of their cell entry pathways can also be expected. Lipid corona formation might also result in (local) lipid oxidation, further increasing the physiologic impact on cells and mammal organisms [19,20].

In this study, two highly amphiphilic glycerophospholipids (GP), POPC (1-palmitoyl-2-oleoyl-sn-glycero-3-phosphocholine) and POPS (1-palmitoyl-2-oleoyl-sn-glycero-3-phospho-L-serine), as well as a combination of both, were investigated. The investigated glycerophospholipids have a similar tail structure, while their head group structure was different [21]. With these different head group structures, the lipids’ charges also differ. POPC is a neutrally charged lipid [22], while POPS is known to be negatively charged [23]. Glycerophospholipids are the main structural lipid components of eukaryotic cell membranes, with phosphatidylcholine representing more than 50% of all cell membrane phospholipids [21]. In line with this, POPC and POPS are described as lipids of the extracellular membrane across all tissue types according to the human metabolome database. POPC is especially found in the placenta, while POPS can be found in the brain, heart, kidney, and liver tissue. Among other lipids, POPC is also an important factor in lung health, as it is a component of pulmonary surfactants crucial for lung health and compliance [24], while POPS is an important factor in apoptosis and enzyme activation [25].

This study aims to understand the formation of lipid coronas with three different lipid compositions and three different MNPP surface charges. In addition, the lipid corona plastic nanoparticles were tested in A549 human lung cells to investigate the impact of these particles on cells. In addition to dermal and enteral uptake, breathing and transmission via the lung tissue is one of the possible routes suggested for MNPP for entering the human body. Even though they are of malignant origin, human A549 lung cancer cells were chosen as a model cell line in this study to address this aspect.

## 2. Materials and Methods

### 2.1. Liposome Preparation by Extrusion

Unilamellar vesicle liposomes were prepared using an extrusion method with modifications from to original protocol [26,27]. Briefly, the liposomes were prepared from 1-palmitoyl-2-oleoyl-glycero-3-phosphocholine (16:0-18:1 PC (POPC); Avanti Polar Lipids, Alabaster, AL, USA) and 1-palmitoyl-2-oleoyl-sn-glycero-3-phospho-L-serine (16:0-18:1 PS (POPS); Avant Polar Lipids, Alabaster, AL, USA) at 1 mM (final concentration). The lipids were used as mono-lipids or combined with POPC:POPS (75 µM:25 µM) to form liposomes. Therefore, the respective amount of lipid was diluted in a glass tube using 2 mL chloroform (Carl Roth, Karlsruhe, Germany) prior to chloroform evaporation in an N_2_-evaporator (TurboVap; Biotage, Uppsala, Sweden) to form a lipid film. The lipid film was rehydrated in an inorganic solvent (i.e., Dulbecco’s phosphate-buffered saline (DPBS); BioWest, Nuaillé, France) and heated to 40 °C in a water bath. After dissolving the lipid film in the inorganic solvent, the solution was either frozen in liquid nitrogen for 1 min or completely frozen, depending on the volume of the inorganic solvent. Afterward, the frozen solution was reheated to 40 °C, and this freeze–thaw process was repeated 5 times. To form unilamellar vesicles of a uniform size, the solution was extruded 13 times through a *Nuclepore* membrane filter with a pore size of 0.1 µm (Whatman products; Cytiva, Marlborough, MA, USA) using a mini extruder (Avanti Polar Lipids, Alabaster, AL, USA) on a 40 °C heating plate. The extruded unilamellar vesicles were stored at 4 °C for a maximum of 3 days.

### 2.2. Interaction of Plastic Particles and Unilamellar Vesicles

**Figure 1:**
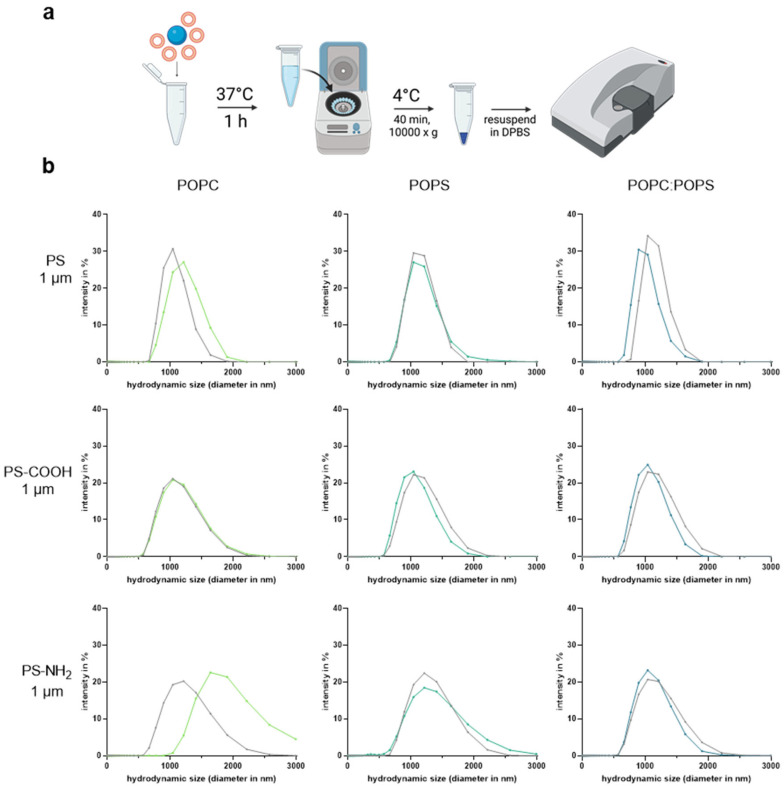
**Dynamic light scattering.** (**a**) interaction protocol of polystyrene MNPP and unilamellar vesicles; (**b**) hydrodynamic size distribution (nm) and intensity weighted (%). Data are presented as the mean of three independent experiments. The plastic particles alone measured via DLS are shown as grey curves, while green (POPC), turquoise (POPS), and blue (POPC:POPS) curves show lipids incubated with respective plastic particles. POPC = 1-palmitoyl-2-oleoyl-glycero-3-phosphocholine; POPS = 1-palmitoyl-2-oleoyl-sn-glycero-3-phospho-L-serine; PS = polystyrene; PS-COOH = carboxylated polystyrene; PS-NH2 = aminated polystyrene. Statistical analysis is from Table 1.

To investigate the effect of the interaction between differently composed unilamellar vesicles and plastic particles, polystyrene (PS) particles (Polysciences Europe, Hirschberg, Germany) with an average size of 1 µm without further modification (PS) or with different surface modifications, such as carboxylation (PS-COOH) or amination (PS-NH_2_), were incubated with unilamellar vesicles. The particles were diluted to 1.25 mg/mL in DPBS and incubated in equal volumes with unilamellar vesicles (1 mM of POPC, POPS, or POPC:POPS at 75 µM:25 µM) for 1 h at 37 °C in a ThermoMixer (Eppendorf, Hamburg, Germany) with an agitation of 300 rpm (Figure 1a). After incubation, suspensions were centrifuged (4 °C) for 45 min at 10,000× *g*, the supernatant was discarded to remove unbound liposomes, and the remaining pellet was resuspended in DPBS.

### 2.3. Dynamic Light Scattering and Zeta Potential

All samples were subjected to dynamic light scattering (DLS). For this, the resuspended particle pellets were diluted in DPBS and measured in DTS1070 cuvettes (Malvern Panalytical, Kassel, Germany) using a Zetasizer Ultra device (Malvern Panalytical, Kassel, Germany). Measurements were performed in technical triplicates both for the hydrodynamic size as well as for the zeta potential. Hydrodynamic size measurements were performed at 25 °C with an equilibration time of 60 s. Zeta potential measurements were performed at 25 °C with an equilibration time of 120 s for a total of 25 runs. The DLS data were exported from the manufacturer’s software ZSXplorer (Malvern Panalytical, Kassel, Germany).

### 2.4. Surface Plasmon Resonance (SPR)

An L1 sensor chip (Cytiva, Marlborough, MA, USA) was coated with unilamellar vesicles consisting of either POPC (1 mM) or a combination of POPC:POPS (75 µM:25 µM). The coating of the sensor chip surfaces was performed in a manual run, starting with three injections of 20 mM 3-[(3-Cholamidopropyl)-dimethylammonio]-1-propansulfonat (CHAPS; Carl Roth, Karlsruhe, Germany) at a flow rate of 10 µL/min for 60 s. After changing the flow rate to 5 µL/min, 1 mM of unilamellar vesicles was injected for 16 min. The unbound unilamellar vesicles were removed by a 60 s injection of 10 mM NaOH Carl Roth, Karlsruhe, Germany) at a 30 µL/min flow rate. To prove that the sensor chip surface was fully coated with the unilamellar vesicles, 0.125 mg/mL bovine serum albumin (BSA; Carl Roth, Karlsruhe, Germany) was injected for 60 s at a flow rate of 10 µL/min. The change in relative units (RU) was investigated after injecting pristine PS into the lipid-coated sensor chip surface. For this, the 1 µm plastic particles were diluted to a concentration of 0.625 mg/mL in DPBS and injected at a flow rate of 10 µL/min for 90 s to allow the interaction with the lipid-coated sensor chip surface. After the experiments, the lipid coating was removed from the sensor chip surface with three 90 s injections of 20 mM CHAPS at a 10 µL/min flow rate.

### 2.5. In Vitro Experiments

Unilamellar vesicles (1 mM) were incubated in equal volumes with three different 1 µm free-labeled plastic particles (PS, PS-COOH, and PS-NH_2_) for 1 h at 37 °C with horizontal agitation (300 rpm). Vesicle mixtures or control solution (DPBS) was then applied to A549 cells (ATTC. CCL-185), which were seeded at 1 × 10^4^ cells in 24-well flat-bottom plates (Thermo Fischer, Dreieich, Germany) for attachment 24 h before treatment in Dulbecco’s Modified Eagle Medium (DMEM; Pan-Biotech, Aidenbach, Germany) with high glucose (4.5 g/L), supplemented with or without 10% fetal calf serum (FCS; Sigma-Aldrich, Taufkirchen, Germany). A549 cells were incubated with 10 µg/mL (final concentration) of the pre-incubated particles for 3 h to 24 h at 37 °C, 5% CO_2_, and 95% humidity. After incubation, the cells were subjected to microscopy or detached using Accutase (BioLegend, Amsterdam, The Netherlands) for flow cytometry.

### 2.6. Flow Cytometry

The effects of MNPP on A549 cells were investigated using flow cytometry (CytoFLEX S; Beckman-Coulter, Krefeld, Germany) 24 h after exposure. The cells were harvested, washed in PBS, and resuspended in 96-well plates with PBS. At least 50,000 cells were acquired per sample. Forward and side-scatter signals were analyzed, and their intensities were quantified using Kaluza Analysis 2.2 software (Beckman-Coulter, Krefeld, Germany). Gating strategies in forward scatter plots were used to determine the percentage of dead cells.

### 2.7. Microscopy Analysis

After exposure of A549 cells to lipid-coated and control MNPP for 24 h, the cells were washed with PBS to remove the remaining particles. Nine fields of view per well were captured using a 20× air objective (NA 0.4; Zeiss, Jena, Germany) and a high-content imaging system (Operetta CLS; PerkinElmer, Hamburg, Germany). After flatfield and brightfield corrections, cells were segmented using digital phase contrast. Algorithm-based quantitative image analysis was performed using *Harmony* 4.9 software (PerkinElmer, Hamburg, Germany) to analyze cell roundness and cell area (µm^2^).

### 2.8. Statistical Analysis

Prism 9.5.1 (GraphPad Software, San Diego, CA, USA) was used for graphs and statistical analyses. If not stated otherwise, data are displayed as mean ± standard deviation (SD). The data sets were tested for normal distribution using the D’Agostino and Pearson tests. For normally distributed data sets, one-way analysis of variance (ANOVA) was used to compare more than two groups. Two groups were tested using a *t*-test. The levels of significance were indicated as follows: α = 0.05 (*), α = 0.01 (**), and α = 0.001 (***).

## 3. Results

### 3.1. Type of Lipid Corona Modulated MNPP Properties

The interaction of differently surface-modified polystyrene MNPP and a unilamellar vesicle model composed of different lipids was investigated using DLS. Changes in hydrodynamic size, as well as zeta potential, were investigated after the incubation of the components (Figure 1b). After the incubation of 1 µm pristine PS particles with a combination of the investigated lipids, POPC:POPS (75 µM:25 µM), a significant change in the polydispersity index (PDI) was measured (Table 1). Several changes in size and PDI were observed, depending on the type of lipid incubated. For the PS-COOH particles, POPS and POPC:POPS significantly increased the PDI. The incubation of PS-NH2 particles with POPC and POPS unilamellar vesicles showed changes in the investigated Z-average as well as in the corresponding PDIs.

**Figure 2:**
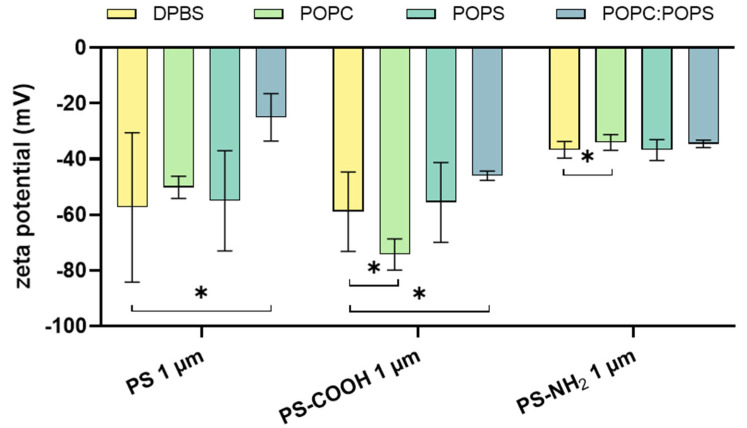
**Zeta potential**. Changes in zeta potential in millivolt (mV) after incubation of pristine (left), carboxylated (middle), and aminated (right) 1 µm plastic particles with the respective unilamellar vesicles. Data are presented as mean ± SD of three independent experiments. Statistical analysis was performed using one-way analysis of variances (ANOVA) with Dunnett’s post hoc test against DPBS (without liposome incubation), and are indicated as follows: * *p* < 0.05; DPBS = Dulbecco’s phosphate-buffered saline; POPC = 1-palmitoyl-2-oleoyl-glycero-3-phosphocholine; POPS = 1-palmitoyl-2-oleoyl-sn-glycero-3-phospho-L-serine; PS = polystyrene; PS-COOH = carboxylated polystyrene; PS-NH_2_ = aminated polystyrene.

**Figure 3:**
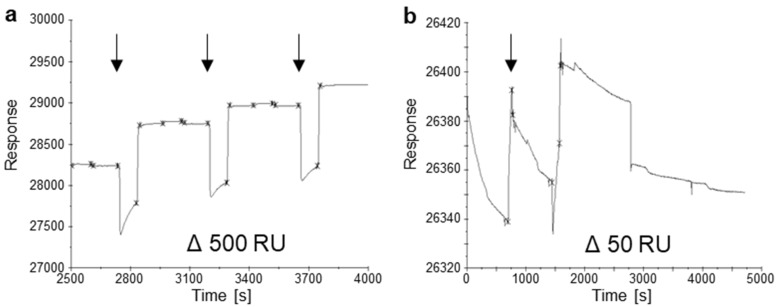
**Surface plasmon resonance responses.** (**a**) Time (s) dependent responses after injection of PS particles into a POPC-coated sensor surface; (**b**) time-dependent responses after the injection of PS particles to a sensor surface coated with POPC:POPS (75 µM:25 µM). RU = response units. Arrows mark the injection timing. Arrows indicate starting of regeneration phases of the sensor.

Subsequent analyses of the suspensions’ zeta potential were performed to investigate the surface charge effects after the exposure of the PS plastic particles to different unilamellar vesicles (Figure 2). Here, the incubation of PS and PS-COOH particles with POPC:POPS liposomes showed a significant increase in the measured zeta potential, whereas the incubation with POPC showed a significant decrease and increase in zeta potential in PS-COOH and PS-NH_2_ MNPP, respectively. Next, the interaction of the PS particles with a lipid-coated gold sensor chip was investigated using surface plasmon resonance (Figure 3). The data indicated a better interaction between the gold sensor chip, which was only coated using POPC, and a change in the relative response units (RU) of around 500 RU after injection of the PS particles. Coating the gold sensor surface with a lipid mixture of POPC:POPS resulted in a response of only 50 RU after the incubation with PS particles, indicating a non-sufficient interaction between the particles and the coated sensor surface. Altogether, these results indicated that the type of lipid corona is decisive in altering MNPP surface characteristics. At the same time, the initial plastic surface charge is also relevant regarding potential modifications by lipid coronas.

### 3.2. Lipid Coronas Affected Nanoparticle Uptake in Cells

**Figure 4:**
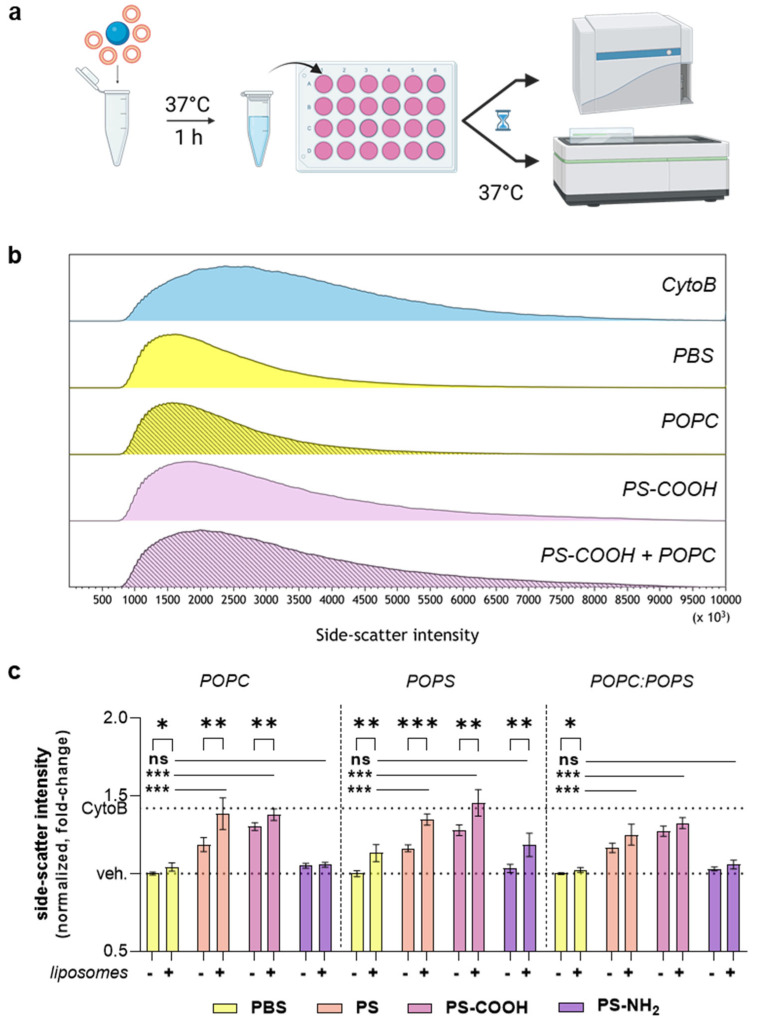
**Cellular plastic particle uptake using flow cytometry.** (**a**) Experimental setup of the pre-incubation of particles with different unilamellar vesicles and subsequent exposure to A549 cells; (**b**) representative side-scatter histograms of cells using flow cytometry; (**c**) side-scatter intensity quantification (%) in conditions with (+) or without (-) liposomes. Data are presented as mean ± SD of three independent experiments. Statistical analysis was performed using one-way analysis of variances (ANOVA) with Dunnett’s post hoc test against DPBS (with liposome incubation) or t-test, comparing the results with and without liposomes, which are indicated as follows: * *p* < 0.05; ** *p* < 0.01; *** *p* < 0.001;. n.s. = non-significant; DPBS = Dulbecco’s phosphate-buffered saline; POPC = 1-palmitoyl-2-oleoyl-glycero-3-phosphocholine; POPS = 1-palmitoyl-2-oleoyl-sn-glycero-3-phospho-L-serine; PS = polystyrene; PS-COOH = carboxylated polystyrene; PS-NH_2_ = aminated polystyrene.

After investigating the effects of the lipid coronas on MNPP surface characteristics, we investigated the biological consequences of the lipid coronas. A549 cells were incubated under nine different MNPP conditions and investigated using flow cytometry 24 h later (Figure 4a). Cytochalasin B (CytoB) was used as a positive control to inhibit actin filament polymerization and cell growth to increase granule formation in cells, leading to increased side-scatter signals (Figure 4b). The side-scatter intensity of 1 µm PS and PS-COOH particles was significantly affected by the presence of POPC and POPS unilamellar vesicles (Figure 4c). Interestingly, no effect was observed with POPC:POPS liposomes.

**Figure 5:**
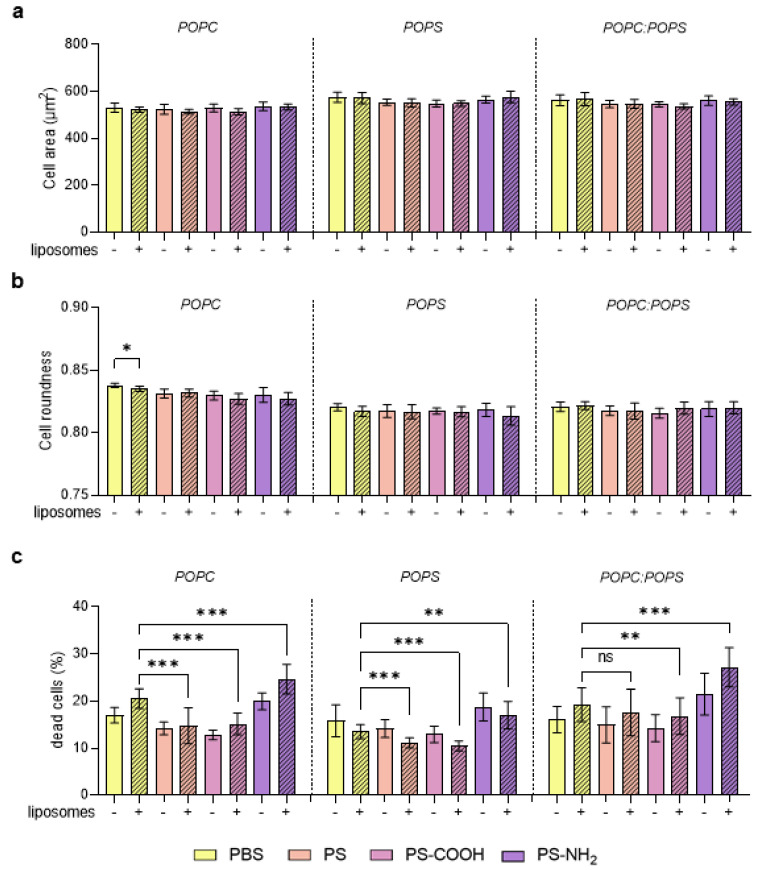
**Cell morphology and viability analysis**. (**a**) Cell areas (µm^2^), (**b**) cell roundness (a.u.), and (**c**) percentage of dead cells in A549 cells incubated with different pre-incubated particles. Data are presented as mean ± SD of three independent experiments. Statistical analysis was performed using ANOVA, comparing results with and without liposomes, which are indicated as follows: * *p* < 0.05; ** *p* < 0.01; *** *p* < 0.001; n.s. = non-significant; DPBS = Dulbecco’s phosphate-buffered saline; POPC = 1-palmitoyl-2-oleoyl-glycero-3-phosphocholine; POPS = 1-palmitoyl-2-oleoyl-sn-glycero-3-phospho-L-serine; PS = polystyrene; PS-COOH = carboxylated polystyrene; PS-NH_2_ = aminated polystyrene.

**Figure A1:**
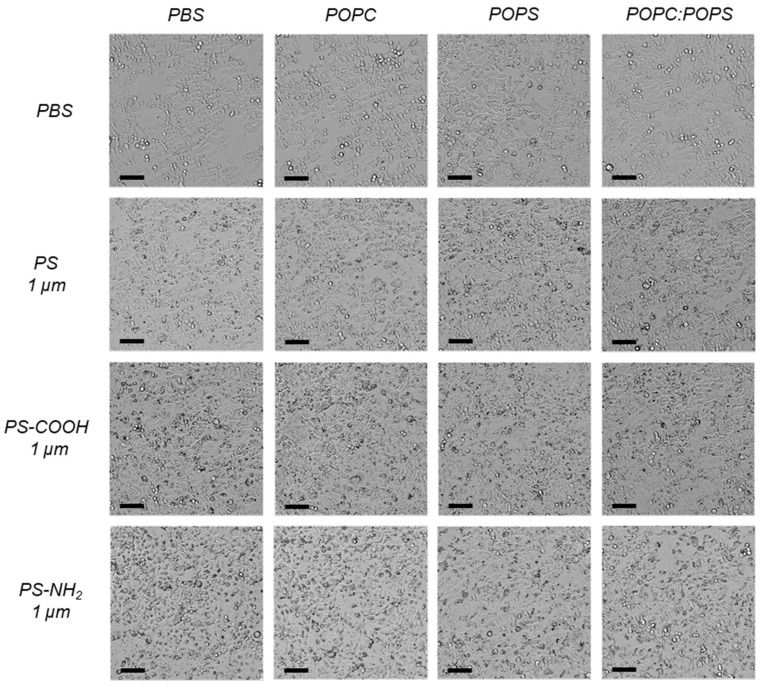
**Representative microscopic A549 cell images.** Images were taken after the exposure of A549 cells to different types of PS particles pre-incubated with or without different types of liposomes. Especially PS-COOH and PS-NH_2_ MNPP incubation with A549 cells increased the appearance of intracellular structures with higher density (dark spots) indicative of particle uptake. This phenomenon can be quantified as an increased side-scatter signal in flow cytometry. The overall cellular size and shape were not affected by MNPP incubation.

Next, we inferred whether MNPP lipid coronas affected the cell morphology (Figure A1). Using algorithm-driven quantitative image analysis, we found no effect of the lipid coronas of MNPP on average cell areas (Figure 5a) and cell roundness (Figure 5b). In contrast, significant differences were observed concerning cell death, which increased when A549 cells were cultured with lipid corona-modified PS-NH_2_ MNPP under all conditions (Figure 5c). However, the PS and PS-COOH mixtures with POPC and POPS showed decreased cell death compared to their control counterparts. In addition, for PS-COOH incubated with POPC:POPS, decreased cytotoxicity was observed, whereas for PS with POPC:POPS, cytotoxicity remained unaltered.

In addition to lipids, proteins are ubiquitous biomolecules present in the body. To this end, we investigated the effect of surplus proteins on the interaction of particles with A549 cells. In this experiment, fluorescently labeled PS particles (PS-NH_2_ 55 nm; PS 190 nm; PS 1040 nm) were pre-incubated with 1 mM POPC unilamellar vesicles for 1 h at 37 °C prior to the exposure of A549 cells to these pre-coated particles for different time points. Under one condition, the cells were washed with PBS, and the cell medium was replaced with a cell culture medium not supplemented with 10% FCS, whereas under the other experimental conditions, the cells were washed with PBS and supplemented with a cell culture medium containing 10% FCS.

**Figure 6:**
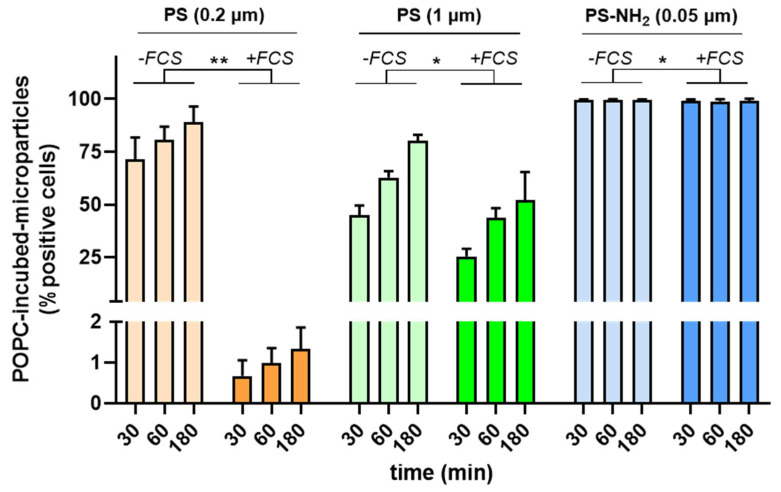
**Contribution of FCS to A549 cell particle uptake.** Measurement of the uptake of plastic particles (pre-incubated with POPC) in A549 cell using flow cytometry and quantified for small (0.2 µm and 1.0 µm) and aminated (55 nm) particles in the presence and absence of FCS quantified at three different time points post-exposure (30 min, 60 min, and 180 min). Data are presented as mean ± SD of three independent experiments. Statistical analysis was performed using multiple ratio-paired t-tests, comparing results with and without FCS, which are indicated as follows: * *p* < 0.05; ** *p* < 0.01; POPC = 1-palmitoyl-2-oleoyl-glycero-3-phosphocholine; PS = polystyrene; PS-NH_2_ = aminated polystyrene.

MNPP uptake was determined by fluorescence using flow cytometry. The presence of FCS led to a significant decrease in the MNPP uptake, independent of their size (Figure 6). The largest effect, however, was observed for the 190 nm particles. Pristine particles (190 nm and 1040 nm) showed increased uptake over time, while positively surface-charged particles (NH_2_) were taken up immediately and were already at a maximum of 30 min post-addition. Altogether, the type of lipid corona significantly affected MNPP uptake into cells, which was the case for the total uptake and its kinetics. Additionally, the particle toxicity was differently affected by the different conditions applied.

## 4. Discussion

Environmental pollution with micro- and nanoplastic particles (MNPP) has evolved over the last decades, infesting the human food chain [8,9,28]. With this rising problem of environmental pollution, the contact of mammalian and human bodies with MNPP is inevitable. Although the interaction of proteins with model plastic particles is studied well [29,30,31], little is known about the interaction of lipids with model polystyrene particles [32]. A corona forms around the particle when plastic particles contact biomolecules like proteins, lipids, and sugars. In the case of well-studied protein coronas, a soft and hard corona is formed around the particles, determining the plastic particles’ fate in organisms [33,34]. Our study investigated the interaction of a unilamellar vesicle model with polystyrene particles modified with different surface chemistries. The model lipids chosen for this study are cell membrane-relevant POPC and POPS [21,35].

Different studies describe the interactions between nanomaterials and lipids in different bodily fluids. Hellstrand and colleagues investigated the interactions of human plasma lipids with nanomaterials and showed that whole lipoprotein complexes are prone to interact with nanomaterials [36]. Our study also suggests an interaction of lipids with polystyrene material, depending on the different surface chemistries. Especially after incubation with suspensions containing a high amount of neutral lipids like POPC, the hydrodynamic size of the positively charged aminated PS particles (PS-NH_2_) increased.

The interaction and the formation of lipid coronas of physiological lipids in mouse serum, like triglycerol and cholesterol, were studied by Lima et al. using PS-COOH particles of different sizes [33]. The study observed that cholesterol binding depends largely on the size of the investigated particles and less on the surface area, whereas triglyceride binding seems to be affected by the surface area of the investigated particles [33]. In contrast, our carboxylated particles did not show increased hydrodynamic sizes independent of the lipid corona type. The reason might be that the structure of the investigated lipids differs from the structures of cholesterol and triglycerides investigated in other studies.

Cholesterol inherits a unique structure with a hydrocarbon tail, a sterol core out of four hydrocarbon rings, and a hydroxyl group, where the hydrocarbon tail and the ring system are non-polar and consequently do not mix with water [37]. Bloodstream transport is, therefore, only possible if water-insoluble lipids like cholesterols are attached to proteins in the bloodstream, the so-called apolipoproteins [38]. Possibly, the attachment of lipids like cholesterol to the lipoproteins is the driving force in the interaction of the lipids with PS particles.

Once particles enter body fluids, corona formation is described as being driven by the reduction of the high surface energy of the particles and is, therefore, a highly dynamic process [33]. Over time, the biocorona composition changes by replacing biomolecules attached early to the particle surface with other biomolecules with a higher affinity to the polymer surface until an equilibrium is reached, which is described as the Vroman effect [39]. In these studies, proteins in the investigated solution, like blood serum, seem to be the driving force in corona formation, during which lipid attachment is more likely an additional effect. Our experiments only investigated solutions containing unilamellar vesicles without a protein admixture in the suspension. The vesicles used in this study were ~100 nm, with hydrophilic head groups located outside the vesicles, whereas the hydrophobic tail structures were centered in a lipidic bilayer. With this stable unilamellar vesicle system, the attachment of the lipids to the particle surface seems to be inhibited.

In the case of positively charged PS-NH_2_ particles, it seems that the driving force of interaction is the charge difference between the particles and the unilamellar vesicles. Theoretically, the unilamellar vesicles composed of POPC should be neutral in charge, but in zeta potential measurements, they show a negative zeta potential. This might be an effect of the suspension medium, in which DPBS, a buffer containing negatively charged phosphates, was used. Potentially, this medium affected the overall charge of the unilamellar vesicles in this suspension and enforced the interaction of the positively charged particles with the unilamellar vesicles containing POPC.

The effect of the buffer medium might also explain why suspensions of only negatively charged unilamellar vesicles, like POPS, did not increase the hydrodynamic size of any of the tested MNPP. Repulsion effects between the unilamellar vesicles and the buffer medium should also be considered, i.e., the particles may not come close enough to the unilamellar vesicles for any interaction to occur. Conversely, the disruption of lipid bilayers is faster and induces a greater leakage with positively charged particles than with negatively charged particles [40]. This effect seems to be due to the surface modification and is independent of the particles’ core material, so the amination of the polystyrene particles can be the reason for their interaction behavior [40,41].

With the POPC:POPS liposome solutions, the PDI and zeta potential changed significantly. PDI changes indicate aggregates formed in the investigated suspension after incubating the liposome suspension with the particles [42]. The significant change in the PDI after the incubation of PS particles with POPC:POPS liposomes, and under some conditions, significant changes in the hydrodynamic size average, indicate that lipid–lipid aggregates might have formed and that an interaction between the particles and the POPC:POPS liposomes is unlikely. This fact can also be assumed since, in the surface plasmon resonance experiments, no interaction between the PS particles and the POPC:POPS-covered sensor chip surface could be measured (Figure 3b). In contrast, the highly significant change in the hydrodynamic size after the incubation of PS-NH_2_ with the POPC liposome suspension without changing the PDI indicates an attachment of the lipids to the investigated particle without forming aggregates. Incubation of POPC:POPS liposomes with PS-NH_2_ showed a significant difference in the average hydrodynamic size. The data also suggested the formation of lipid–lipid aggregates in the PDI.

Zeta potential change is an important measurement to characterize the suspension’s stability and investigate the surface functionality of particle dispersions [43]. The resulting zeta potential occurs due to the interaction of ions solubilized in the measuring buffer with the functional groups on the particles’ surface. Suspensions with zeta potentials ranging between −30 mV and +30 mV are considered stable due to the sufficient repulsive force in the system to avoid aggregate formation in the solution [44]. The change in zeta potential after the incubation of the unilamellar vesicles consisting of POPC:POPS with PS particles indicates that the solution stabilizes in the presence of POPC:POPS liposomes. In a suspension system considered stable, repulsion occurs between the components, so an interaction seems very unlikely.

The significant decrease in zeta potential after adding POPC liposomes to PS-COOH particles suggests that the suspension is no longer stable and is more prone to form aggregates. Yet, the formation of aggregates was not observed in the analysis of hydrodynamic sizes. We assume that due to repulsion effects, no aggregates and no interaction of the particles and the added liposomes were formed due to the same surface charges of the components. While the interaction of PS particles with POPC liposomes showed changes in neither the hydrodynamic size nor the PDI, an interaction of these components was observed in SPR analysis. This might be due to a larger lipid interaction surface and, therefore, more attraction of the particles to the lipid-coated sensor surface than to the liposomes.

A549 cells responded differently to MNPP, with and without lipid corona. Cytotoxicity was significantly altered, as previously shown by others [45,46] and ourselves [47], and PS-NH_2_ showed more pronounced effects. Interestingly, we presume that particle size may be a distinguishing factor in aminated particle cytotoxicity. Smaller particles, such as 55 nm, are readily cytotoxic, as previously demonstrated [47], while larger particles, such as the 1 µm PS-NH_2_ particles used in our study, were cytotoxic, but not of the same magnitude. This was potentially due to the limited uptake of positively charged particles at that size, as suggested by the side-scatter quantification in our study (Figure 4c). Interestingly, POPC lipid addition to these particles did not affect the uptake rates either. This is notable since this condition showed the most significant size change effect in our study (Figure 1b). This can be explained by the size of the pre-incubated particles, which are too large to interact with the cells [48]. Moreover, POPC incubation seemed to mask the uptake-increasing effects of POPS on PS-NH_2_, as incubation with POPC:POPS PS-NH_2_ did not differ from particles without adding lipids.

Generally, pre-incubated PS and PS-COOH particles seemed to interact more in our A549 cell model. This is concerning because environmental particles that were ingested or inhaled also had a kind of “pre-coating” with different biomolecules before or while entering the human food chain [49]. This corona formation around the environmentally relevant particles can also alter the interaction behavior of the particles within cells and organisms. The particles can also transport chemicals to mammalian organisms, such as ethane (DDT), polychlorinated biphenyls (PCB), and so-called endocrine disruptors, potentially affecting the hormone systems [50].

Our study could not provide direct evidence of lipid corona formation on MNPP. Nevertheless, DLS experiments have shown increasing polystyrene particle sizes following incubation with lipids, suggesting the latter’s coating on the plastic particles. Alternatively, future studies could perform mass spectrometry analysis to provide unambiguous evidence of lipid attachment to the MNPP. The biological relevance of our study is also potentially hampered by the fact that MNPP modification by, e.g., protein or lipid coronas, would occur already before entering the cells. This could be due to, for example, the mucus that covers the epithelium. In addition, major MNPP modifications may also be formed only after uptake into the circulatory system. Future in vivo trials may reveal the presence and relevance of MNPP modifications via the respiratory route.

## 5. Conclusions

The interaction between lipids and plastic particles was studied using a liposome model, and polystyrene (PS) particles were subjected to DLS, zeta potential measurements, and SPR analysis. The type of lipid and plastic particle surface charge also affected the uptake and toxicity of human A549 lung cells differently. In future studies, polymethyl methacrylate (PMMA), polyethylene terephthalate (PET), and polypropylene (PP) particles in the sub-micron range should be studied, and for PS model particles, both dynamics and lipid oxidation should be investigated via high-resolution mass spectrometry. These studies are needed to prove the dependencies of cell interactions on lipid charge, nanoparticle material (PMMA/PVC), and surface modification.

## Figures and Tables

**Table 1:** **Dynamic light scattering quantification.** Polydispersity index (PDI) and Z-average (in nm) measurements of polystyrene MNPP and unilamellar vesicles. Data are presented as mean ± SD of three independent experiments. DPBS = Dulbecco’s phosphate-buffered saline; POPC = 1-palmitoyl-2-oleoyl-glycero-3-phosphocholine; POPS = 1-palmitoyl-2-oleoyl-sn-glycero-3-phospho-L-serine; PS = polystyrene; PS-COOH = carboxylated polystyrene; PS-NH_2_ = aminated polystyrene. Significant differences from the DPBS control groups are indicated in **bold** (* *p* < 0.05; ** *p* < 0.01; *** *p* < 0.001; n.s. = non-significant).

		PS	PS-COOH	PS-NH_2_
DPBS	PDI	0.42 ± 0.05	0.21 ± 0.05	0.19 ± 0.04
POPC	PDI	0.41 ± 0.15 ^n.s.^	0.20 ± 0.10 ^n.s.^	0.21 ± 0.10 ^n.s.^
POPS	PDI	0.42 ± 0.06 ^n.s.^	0.26 ± 0.05 **	0.28 ± 0.10 *
POPC:POPS	PDI	0.55 ± 0.12 **	0.27 ± 0.05 *	0.24 ± 0.03 **
DPBS	Z-average	1753 ± 224.10	1193 ± 45.08	1247 ± 38.26
POPC	Z-average	1878 ± 108.70 ^n.s.^	1193 ± 57.60 ^n.s.^	2365 ± 552.7 ***
POPS	Z-average	1785 ± 131.90 ^n.s.^	1195 ± 24.67 ^n.s.^	1414 ± 329.9 ^n.s.^
POPC:POPS	Z-average	1937 ± 193.70 *	1226 ± 35.87 ^n.s.^	1188 ± 16.00 **

## Data Availability

The underlying data of this study are available from the corresponding author upon reasonable request.
